# Associations Among Hip Structure, Bone Mineral Density, and Strength Vary With External Bone Size in White Women

**DOI:** 10.1002/jbm4.10715

**Published:** 2023-01-16

**Authors:** Karl J Jepsen, Erin MR Bigelow, Michael A Casden, Robert W Goulet, Kathryn Kennedy, Samantha Hertz, Chandan Kadur, Bonnie T Nolan, Kerry Richards‐McCullough, Steffenie Merillat, Carrie A Karvonen‐Gutierrez, Gregory Clines, Todd L Bredbenner

**Affiliations:** ^1^ Department of Orthopaedic Surgery (Medical School) and Department of Epidemiology (Public Health) University of Michigan Ann Arbor MI USA; ^2^ Department of Biomedical Engineering Marquette University Milwaukee WI USA; ^3^ Endocrinology VA Medical Center Ann Arbor MI USA; ^4^ Department of Mechanical and Aerospace Engineering University of Colorado Colorado Springs Colorado Springs CO USA

**Keywords:** BONE MINERAL DENSITY, EXTERNAL SIZE, POPULATION HETEROGENEITY, PROXIMAL FEMUR, STRENGTH, STRUCTURE

## Abstract

Bone mineral density (BMD) is heavily relied upon to reflect structural changes affecting hip strength and fracture risk. Strong correlations between BMD and strength are needed to provide confidence that structural changes are reflected in BMD and, in turn, strength. This study investigated how variation in bone structure gives rise to variation in BMD and strength and tested whether these associations differ with external bone size. Cadaveric proximal femurs (*n* = 30, White women, 36–89+ years) were imaged using nanocomputed tomography (nano‐CT) and loaded in a sideways fall configuration to assess bone strength and brittleness. Bone voxels within the nano‐CT images were projected onto a plane to create pseudo dual‐energy X‐ray absorptiometry (pseudo‐DXA) images consistent with a clinical DXA scan. A validation study using 19 samples confirmed pseudo‐DXA measures correlated significantly with those measured from a commercially available DXA system, including bone mineral content (BMC) (*R* ^*2*^ = 0.95), area (*R* ^*2*^ = 0.58), and BMD (*R* ^*2*^ = 0.92). BMD–strength associations were conducted using multivariate linear regression analyses with the samples divided into narrow and wide groups by pseudo‐DXA area. Nearly 80% of the variation in strength was explained by age, body weight, and pseudo‐DXA BMD for the narrow subgroup. Including additional structural or density distribution information in regression models only modestly improved the correlations. In contrast, age, body weight, and pseudo‐DXA BMD explained only half of the variation in strength for the wide subgroup. Including bone density distribution or structural details did not improve the correlations, but including post‐yield deflection (PYD), a measure of bone material brittleness, did increase the coefficient of determination to more than 70% for the wide subgroup. This outcome suggested material level effects play an important role in the strength of wide femoral necks. Thus, the associations among structure, BMD, and strength differed with external bone size, providing evidence that structure–function relationships may be improved by judiciously sorting study cohorts into subgroups. © 2022 The Authors. *JBMR Plus* published by Wiley Periodicals LLC on behalf of American Society for Bone and Mineral Research.

## Graphical abstract

Strength and the associated bone density distribution images vary widely for any given BMD value.

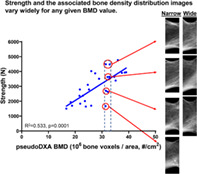

## Introduction

Hip fractures are associated with loss of independence, chronic pain, disability, decline in quality of living, increased mortality, and substantial economic costs.^(^ ^1^, ^2^ ^)^ Reducing hip fracture incidence remains a major public health concern,^(^ ^1^, ^3^, ^4^, ^5^ ^)^ as fracture rates, once declining,^(^ ^2^, ^6^, ^7^ ^)^ have plateaued^(^ ^8^ ^)^ and are expected to increase worldwide attributable in part to increasing numbers of individuals older than 65 years of age.^(^ ^9^, ^10^ ^)^ Individuals at increased risk of fracturing a hip are identified primarily using femoral neck (FN) areal bone mineral density (BMD) as assessed by dual‐energy X‐ray absorptiometry (DXA). However, the osteoporosis threshold criterion (*T*‐score ≤ −2.5) identifies only half of the individuals who fracture,^(^ ^11^, ^12^, ^13^, ^14^ ^)^ resulting in large numbers of individuals who may have benefited from fracture‐reducing treatments. Better understanding of the association between BMD and bone strength may help refine the clinical use of this technology in identifying individuals at high risk of fracturing.

Clinically, BMD is used to monitor bone strength decline with the assumption that a reduction in BMD reflects structural changes that compromise strength. However, the structural variations that give rise to the variation in DXA parameters (ie, bone mineral content [BMC], area, BMD) and strength are not fully understood. Prior work has related bone structure with BMD at the image resolution of clinical CT or MRI,^(^ ^15^, ^16^, ^17^ ^)^ consistent with the use of these technologies on living humans. Only a few studies related BMD to measures of trabecular architecture^(^ ^18^, ^19^, ^20^ ^)^; these studies were conducted on cadaveric tissue to enable image acquisition at the higher resolutions required for microstructural quantification. Thus, despite the prevalent use of DXA BMD, we do not have a full understanding of how variation in bone structure contributes to BMD and, in turn, whole bone strength.

**Fig. 1:**
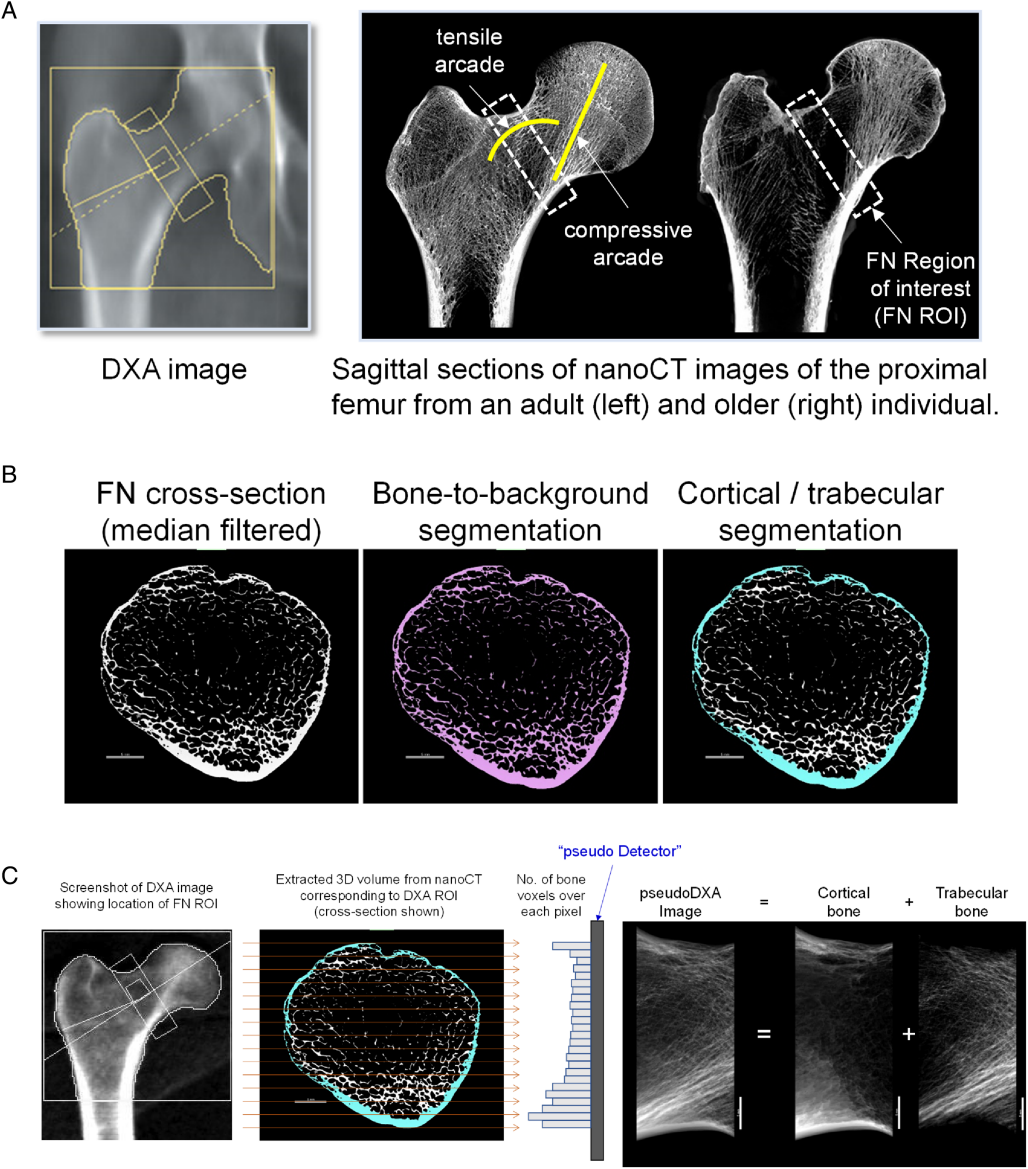
Schematic showing (*A*) the placement of the standard region of interest for a DXA image of a proximal femur and the corresponding region of interest for sagittal sections from nano‐CT images of the proximal femurs of a younger (left) and older (right) donor. (*B*) A femoral neck cross section shows the outcomes of the bone‐to‐background and cortical‐trabecular segmentations. (*C*) The pseudo‐DXA image conveys the distribution of image intensity, reflecting the number of bone voxels, for cortical bone alone, trabecular bone alone, and the combined cortical and trabecular bone. The trabecular arcades and cortices are indicated for orientation purposes.

Hip BMD is calculated as the ratio of BMC to bone area for a standardized FN region of interest (ROI). Bone area provides a measure proportional to outer bone size, whereas BMC provides a measure proportional to the amount of bone tissue.^(^ ^21^ ^)^ Studies investigating how DXA parameters relate to bone structure focused primarily on the association between BMC and the proportion of cortical and trabecular bone. FN BMC is thought to reflect a fixed 60:40 ratio for cortical/trabecular mass.^(^ ^22^, ^23^, ^24^ ^)^ However, the femoral neck is constructed with multiple, interacting trabecular arcades traversing the FN ROI^(^ ^25^, ^26^ ^)^ and varying proportions of cortical and trabecular bone along the length^(^ ^22^, ^27^ ^)^ and around the circumference^(^ ^28^, ^29^ ^)^ (Fig. 1*A*). The proportion of cortical and trabecular bone also varies among individuals depending on outer bone size, with wider femoral necks showing greater cortical area on an absolute basis but lower area on a relative basis compared with narrower femoral necks.^(^ ^30^ ^)^ Critically, BMD did not differ between the narrow and wide groups, suggesting different bone structures give rise to similar BMD values. These studies suggested BMD is not uniquely related to bone structure, which has the potential to complicate the association between BMD and strength.

The primary goal of this study was to determine how variation in bone structure correlates with FN DXA parameters (BMC, area, BMD) and whole bone strength. We tested the hypothesis that associations between DXA parameters and strength depend on outer bone size. To accomplish these goals, bone mineral distribution maps, or “pseudo‐DXA” images, were generated from high‐resolution nano‐computed tomography (nano‐CT) images of cadaveric proximal femurs corresponding to the region assessed during a routine hip DXA scan. The pseudo‐DXA images provided full access to the bone mineral distributions, which allowed us to study the associations among DXA parameters and the interindividual variation in bone structure and strength.

## Materials and Methods

### Samples

Unfixed cadaveric proximal femurs (*n* = 30) from White female donors (36–89+ years of age) with no known musculoskeletal pathologies were obtained from the University of Michigan Anatomical Donations program (Ann Arbor, MI, USA), Science Care (Phoenix, AZ, USA), and Anatomy Gifts Registry (Hanover, MD, USA). Human tissue use and handling procedures were approved by the University of Michigan Institutional Biosafety Committee (IBC), and this study was declared exempt by the Institutional Review Board (IRB). Femurs were cut transversely 16.5 cm distal to the superior aspect of the femoral head. The femoral shaft was embedded in a 5‐cm‐square aluminum channel filled with acrylic resin (Ortho‐Jet BCA, Lang Dental, Wheeling, IL, USA) using a custom alignment fixture so the femoral neck was oriented at 15 degrees of internal rotation relative to the embedding block faces. The embedding block thus allowed for consistent sample orientation.

### Dual‐energy X‐ray absorptiometry

A total of 19 of the samples were used as a pseudo‐DXA image validation subgroup and prepared for DXA scanning. The proximal femurs with embedding blocks were placed within two clear plastic, sealable bags. Biosafety level 2 (BSL2) safety protocols necessitated minimizing the potential for fluid leaks; as such, the samples could not be imaged while immersed in water. Instead, each sample was scanned while covered with approximately 2 inches of rice to simulate muscle.^(^ ^31^ ^)^ The samples were held in place by the embedding blocks using a custom‐built fixture that allowed scanning to be conducted at any angle. All scanning angles were confirmed using a digital inclinometer with a resolution of 0.1 degrees. DXA images of the potted proximal femurs were acquired on a QDR Discovery A Bone Densitometer (Hologic Inc., Marlborough, MA, USA) using a standard clinical protocol. Sagittal images were acquired for each sample with the femoral neck internally rotated 15 degrees, consistent with clinical imaging practices. FN BMC, area, and BMD were calculated using the manufacturer's software.

### Nano‐computed tomography imaging

High‐resolution 3D images of all 30 proximal femurs were obtained using a nano‐CT system (nanotom‐m, phoenix|x‐ray, Wunstorf, Germany) with the following parameters^(^ ^32^ ^)^: 27 μm voxel size, 110 kV, 200 μA, 0.762 mm aluminum filter. Calibration phantoms containing air, water and hydroxyapatite mimickers (1.69 mg/cc; Gammex, Middleton, WI, USA) were included in each scan. Full‐image volumes were reconstructed using datos|x software (GE Inspection Technologies, LP, Skaneateles, NY, USA), and gray values were converted to Hounsfield units using the calibration phantoms.

Dragonfly software (version 2021.1.0.977; Object Research Systems Inc, Montreal, Canada) was used to create a FN volume of interest (VOI) corresponding to a standard DXA ROI from each nano‐CT volume. The nano‐CT volumes were rotated 15 degrees to remove the anteversion, thereby aligning the FN axis in parallel with the *x‐y* plane of the nano‐CT scanner. A 15‐mm‐thick VOI perpendicular to the FN axis was extracted, with one corner placed at the inflection point between the femoral neck and the greater trochanter on the superior side of the bone consistent with the ROI placement used by the Hologic scanner. A repeatability study was performed to confirm consistent VOI extraction by a single individual performing this method three times each on five samples. The intraclass correlation coefficient (ICC 3,1) and the 95% confidence intervals (CI) were calculated based on a single rater, consistency, two‐way mixed effects model. Reliability was determined based on the 95% CI of the ICC estimate, where values grouped by less than 0.5, 0.5–0.75, 0.75–0.9, and greater than 0.9 indicate poor, moderate, good, and excellent reliability, respectively. Reliability was excellent for area ICC = 0.9997 (95% CI 0.9987–0.9999) and BMC ICC = 0.988 (95% CI 0.9433–0.9987). A validated fully convolutional neural network (FCNN) was used to segment bone from background (DICE coefficient = 0.983 ± 0.016). A second validated FCNN was used to segment cortical from trabecular bone (Dice coefficient = 0.977 ± 0.023) to generate cortical and trabecular subvolumes for structural quantification. The boundary between cortical and trabecular bone was defined in accordance with prior studies^(^ ^28^, ^29^ ^)^; examples are shown in Fig. 1*B*. The segmented image files were evaluated for thresholding quality and cortical‐trabecular segmentation. Erroneously identified voxels were manually corrected. The structural measures assessed from the 3D nano‐CT volumes included trabecular volume fraction (BV/TV), trabecular thickness (Tb.Th), average cortical thickness (Ct.Th), total cross‐sectional area (Tt.Ar), and cortical area (Ct.Ar).

### Pseudo‐DXA images

A 2D bone density mapping was generated for each nano‐CT VOI (*n* = 30) by projecting all bone voxels onto a planar FN ROI. The anatomical location, orientation, and width of the FN ROI were consistent with the Hologic DXA scanner used for the validation study. The 2D mapping was called the pseudo‐DXA image (Fig. 1*C*). This analysis focused exclusively on the number of bone voxels, since the X‐ray attenuation giving rise to BMC and the associated image reflects primarily the mineral component.^(^ ^33^ ^)^ The contributions of marrow and X‐ray scattering to the pseudo‐DXA image were not assessed, as this initial study focused on the relative contributions of cortical and trabecular tissues to the DXA parameters.

The parameters calculated from the pseudo‐DXA images included the ROI area (pseudo‐DXA area), number of cortical bone voxels, number of trabecular bone voxels, total number of bone voxels (pseudo‐DXA BMC), and total number of bone voxels/area (pseudo‐DXA BMD). The numbers of cortical and trabecular bone voxels above (superior) and below (inferior) the FN ROI midline were also calculated. The average FN width and minimum FN width were measured directly from the pseudo‐DXA image. All terms preceded by pseudo‐DXA designate traits defined from the projected nano‐CT images, whereas terms preceded by DXA refer to traits determined by the DXA system.

### Mechanical testing

Proximal femurs (*n* = 30) were loaded to failure on an Instron 8511 (Instron, Inc., Norwood, MA, USA) in a fall‐to‐the‐side configuration to collect measures of bone strength, as previously described.^(^ ^34^ ^)^ Samples were oriented so the shaft was 10 degrees relative to a horizontal plane and the femoral neck axis was internally rotated 15 degrees, consistent with the loading configuration experienced during a sideways fall and with prior studies.^(^ ^35^ ^)^ The load was applied through a metal acetabular cup that was custom fitted to the sample based on the femoral head diameter. A custom polyester putty‐filled (Bondo, 3M, Inc., St. Paul, MN, USA) pad was used to distribute load to the greater trochanter. Bones were preloaded to 100 N to ensure proper seating of the sample and fixtures and then loaded to failure at a displacement rate of 100 mm/s. Maximum load (N), stiffness (N/mm), yield load (N), and post‐yield displacement (PYD, mm) were calculated from the load–displacement curves. The yield point was defined as the location where a 10% reduction in the stiffness regression crossed the load‐displacement curve. A validation study determined the deflection attributable to the load cell and Bondo pads was 0.04 mm (0.02–0.1 mm), which accounted for 0.96% (0.56%–2.2%) of the total displacement of the fractured femurs. For simplicity, maximum load was used synonymously with whole bone strength. An error in data acquisition prevented the collection of strength data for 1 sample.

### Statistical analysis

For clarification of the sample size, the DXA validation study was conducted on a subset of the sample cohort; the validation sample size was limited based on the availability of the system, which was actively being used to acquire bone density data on living humans for numerous independently funded studies. Data are expressed as mean ± standard deviation, unless otherwise indicated. Variables were tested for normality (Shapiro–Wilk test, *p* < 0.05) and transformed using log or square root, if necessary. Three primary analyses were conducted. First, linear regression analysis was used to examine the relationship between the pseudo‐DXA parameters and standard DXA parameters. We also determined the extent to which variation in pseudo‐DXA BMC arose from the proportion of cortical and trabecular bone in the superior and inferior halves and how these individual contributors correlated with age. Second, we tested if the proportion of cortical and trabecular bone varied with external bone size. Samples were sorted into narrow (*n* = 15) and wide (*n* = 15) subgroups using pseudo‐DXA area. This sorting was conducted without height adjustment because height information was not available for all donors. Linear regression analysis was used to relate pseudo‐DXA BMC and the number of cortical bone voxels, the number of trabecular bone voxels, and the proportion of cortical bone voxels. ANCOVA was used to test whether the slope and *y*‐intercepts differed between narrow and wide subgroups.

Third, predictors of bone strength were identified using multivariate linear regression analysis. The analysis was conducted using the full data set and with the data divided into narrow and wide subgroups. Regression models started with predictors that are available clinically (model 1: age, body weight, BMD), then proceeded to replace general predictors with parameters that provide progressively more refined information. For example, in model 2, pseudo‐DXA BMD was replaced with its constituents’ pseudo‐DXA area and pseudo‐DXA BMC. In model 3, pseudo‐DXA BMC was replaced with the amount of bone in the superior and inferior halves of the FN. In model 4, the amount of bone (ie, number of voxels) in the superior and inferior halves was segmented into the number of cortical and trabecular bone voxels in the superior and inferior halves of the FN. Finally, model 5 included PYD, a measure reflecting material‐level brittleness, to the regression analysis, based on prior work showing the dependence of whole bone strength on PYD.^(^ ^34^, ^36^ ^)^ Although samples were sorted based on pseudo‐DXA area, pseudo‐DXA area was included in models 2 to 5 to generate consistent regression models across the groupings (ie, all samples, narrow, wide) and because this measure remains a continuous variable despite the sorting. The log of body weight was used in the regression analyses because body weight failed the Shapiro–Wilk normality test. The square root of PYD was used in the regression analysis because PYD failed the normality test. Variance inflation factors (VIF) were calculated for each model. For models showing one or more variables with VIF >10, the multiple variable regression analysis was repeated with the variable having the maximum VIF removed. This process resulted in VIF <10 for all variables. All bivariate linear regression analyses were conducted using GraphPad Prism (v. 9.1.0; GraphPad Software, La Jolla, CA, USA). Linear regression modeling was conducted using SPSS (IBM SPSS Statistics, v. 27, Armonk, NY, USA).

Because actual DXA images were acquired for only 19 samples and pseudo‐DXA images were generated for 30 samples, it was not possible to test the impact of discordance in the assignment to narrow versus wide subgroups when rank‐ordering the samples using DXA area versus pseudo‐DXA area. When sorting the 19 samples used in the validation study by actual DXA area, there was some discordance between assignment to the narrow versus wide subgroup, and most of the discordant assignments were near the boundary dividing the rank‐ordered samples into narrow and wide subgroups. Because the validation cohort was too small to repeat the analyses while removing discordant samples, we conducted a sensitivity analysis that removed a progressively larger number of samples from the middle of the pseudo‐DXA area rank‐ordered samples. This sensitivity analysis tested whether the major outcomes depended on assignment to the narrow or wide subgroup by removing the middle 4 (2 narrow and 2 wide excluded), middle 8 (4 narrow and 4 wide excluded), and middle 12 (6 narrow and 6 wide excluded) samples. We repeated the analysis of the association between pseudo‐BMC and the proportion of cortical and trabecular bone and the multivariate regression analysis.

## Results

### Correlations between pseudo‐DXA and DXA parameters

**Fig. 2:**
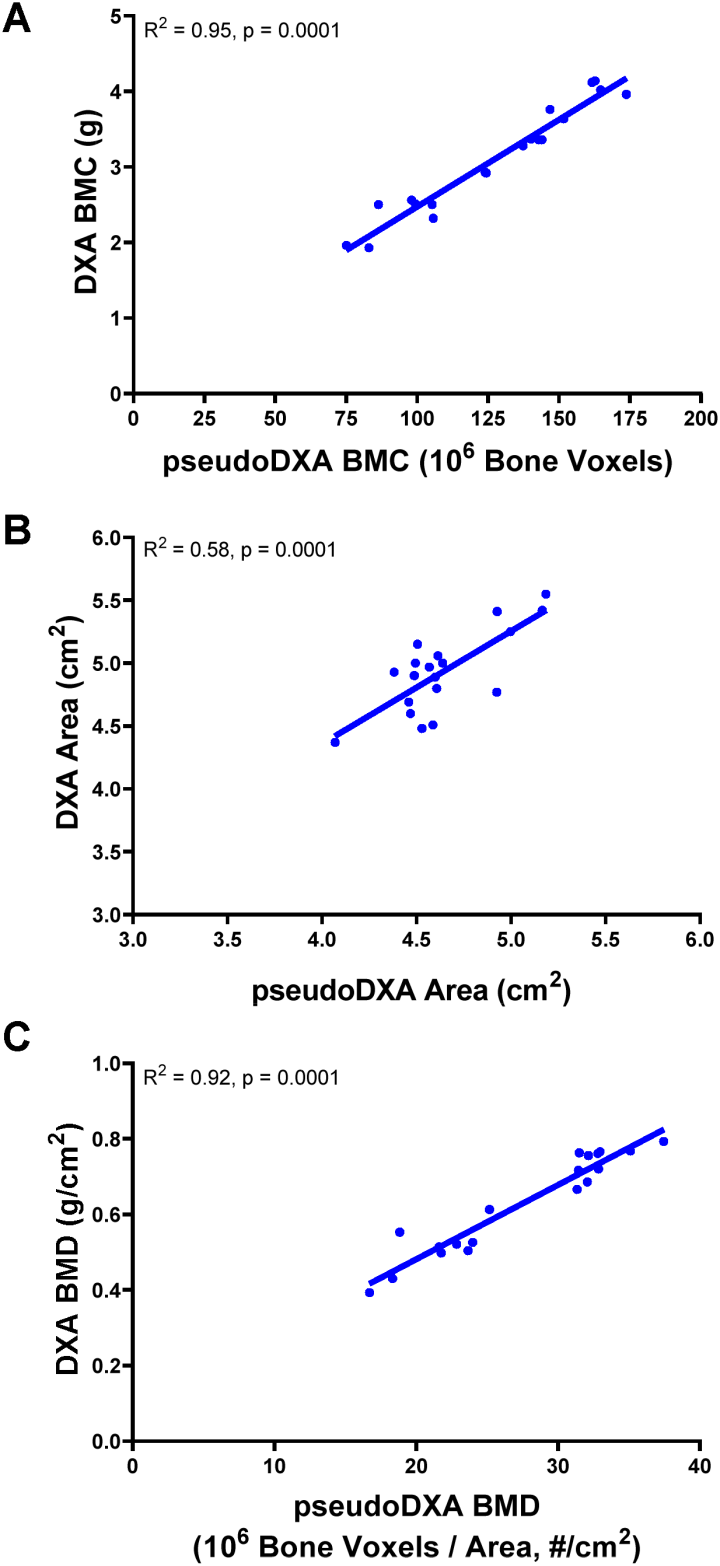
Linear regression analyses show strong correlations between (*A*) pseudo‐DXA area and DXA area, (*B*) pseudo‐DXA bone mineral content (BMC; total number of bone voxels) and DXA BMC, and (*C*) pseudo‐DXA bone mineral density (BMD; total bone voxels/area) and DXA BMD. The linear regressions included 19 samples.

Linear regression analyses were conducted to assess the relationship between pseudo‐DXA parameters and standard DXA parameters for the 19 proximal femurs scanned on both the nano‐CT and Hologic DXA systems (Fig. 2). Significant correlations were found between pseudo‐DXA BMC (total number of bone voxels) and DXA BMC (*R* ^2^ = 0.947, *p* = 0.0001), pseudo‐DXA area and DXA area (*R* ^2^ = 0.581, *p* = 0.0001), and pseudo‐DXA BMD (total number of bone voxels normalized by the pseudo‐DXA area) and DXA BMD (*R* ^2^ = 0.919, *p* = 0.0001). The slope of the pseudo‐DXA area – DXA area regression was 0.90 (95% CI 0.51–1.29), but the *y*‐intercept was 0.78 (95% CI −1.03–2.58), indicating the pseudo‐DXA area underestimated the area determined from the DXA system. These significant correlations demonstrated high correspondence between the pseudo‐DXA and standard DXA parameters, confirming the rigor of our methodology. A preliminary study comparing DXA parameters with 1 inch versus 2 inches of rice confirmed the amount of rice had no impact on the outcome parameters (FN area, *p* = 0.348; FN BMC, *p* = 0.499, FN BMD, *p* = 0.302; paired *t* tests).

### Associations among pseudo‐DXA parameters and bone structure

Significant correlations (Table 1) were found between pseudo‐DXA area and measures of outer bone size including FN width (*R* ^2^ = 0.935, *p* = 0.0001) and total cross‐sectional area (*R* ^2^ = 0.523, *p* = 0.0001). Pseudo‐DXA BMC correlated significantly with measures of the amount of bone, including Ct.Ar (*R* ^2^ = 0.706, *p* = 0.0001), Ct.Th (*R* ^2^ = 0.509, *p* = 0.0001), and trabecular BV/TV (*R* ^2^ = 0.969, *p* = 0.0001). Pseudo‐DXA BMD showed similar significant correlations with these structural measures.

**Table 1:** Correlation Analysis Among Pseudo‐DXA Parameters and Structural Measures Assessed From the Nano‐CT (*n* = 30; *R* ^2^ Values Shown)

Structural measure	Pseudo‐DXA area	Pseudo‐DXA BMC	Pseudo‐DXA BMD
Min FN width	**0.935**	**0.185**	0.035
Tt.Ar	**0.523**	0.003	0.025
Ct.Ar	0.042	**0.706**	**0.644**
Ct.Th	**0.500**	**0.509**	**0.556**
Trabecular BV/TV	0.046	**0.969**	**0.839**

*Note*: Significant correlations (*p* < 0.05) indicated in bold.

Abbreviations: Min FN width = width at the minimum femoral neck location; Tt.Ar = cross‐sectional total area averaged over region of interest; Ct.Ar = cross‐sectional cortical area averaged over region of interest; Ct.Th = average cortical thickness; BV/TV = bone volume fraction (bone volume divided by total volume contained with the subendosteal boundary); BMC = bone mineral content; BMD = bone mineral density.

### Contributions of regional bone density to BMC

**Fig. 3:**
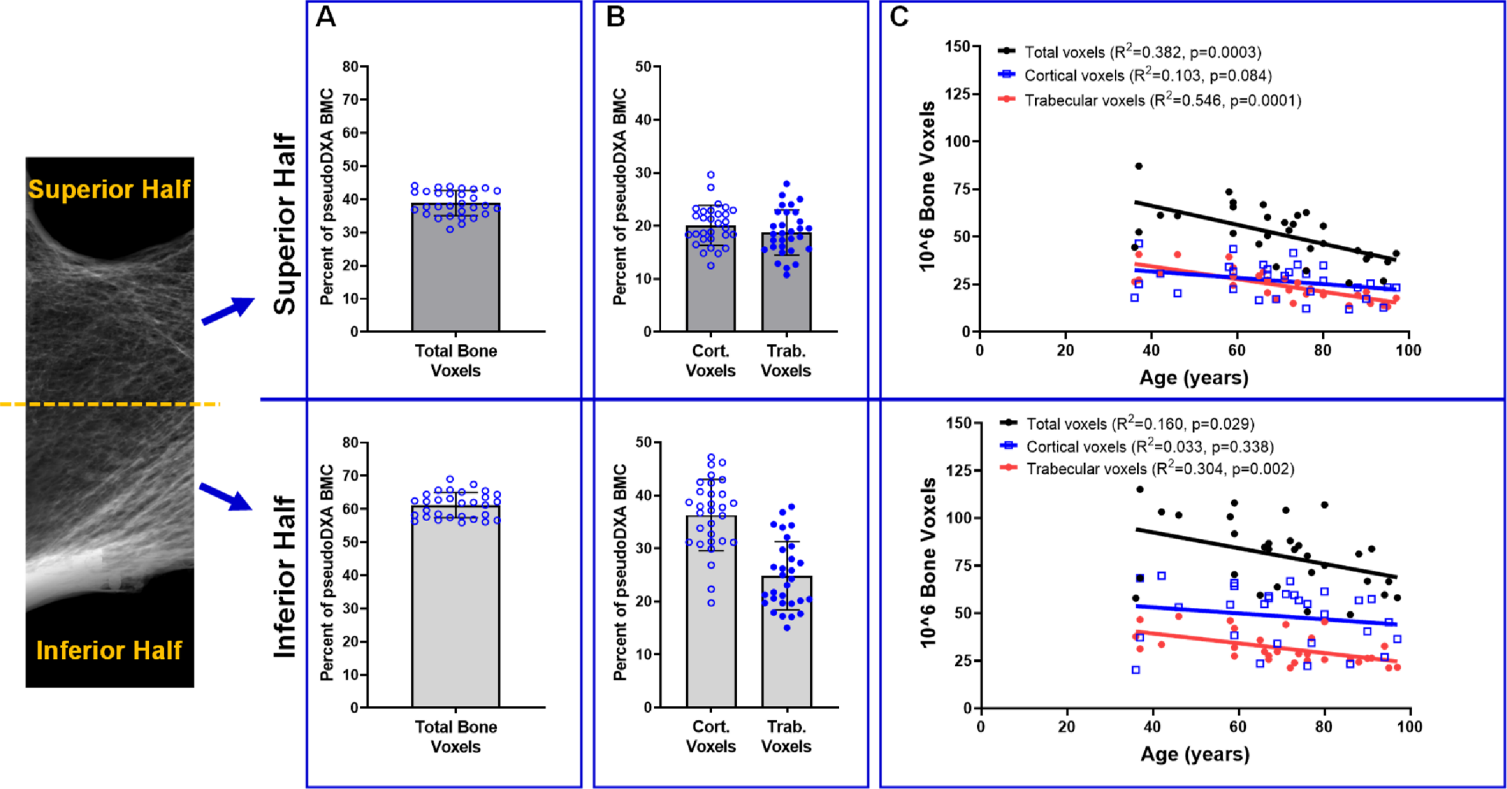
Distribution of (*A*) total and (*B*) cortical and trabecular voxels within the superior and inferior halves of the femoral neck. (*C*) The total number of voxels and the number of cortical and trabecular voxels in the superior and inferior regions show different associations with age based on linear regression analysis. The analyses included 30 samples. Data shown in (*A, B*) are mean and standard deviation.

The number of cortical and trabecular voxels were calculated for the superior and inferior halves of the FN ROI and related to pseudo‐DXA BMC to quantify the extent to which the nonuniform distribution of bone density contributes to the variation in BMC. Splitting the pseudo‐DXA image into superior and inferior halves based on a simple midline transection resulted in 50.5 ± 1.2% of the FN area being assigned to the superior half and 49.5 ± 1.2% being assigned to the inferior half. The total number of bone voxels in the superior and inferior halves comprised 38.8 ± 3.8% (mean ± standard deviation) and 61.2 ± 3.8% of the pseudo‐DXA BMC, respectively (Fig. 3). For the superior half, 20.1 ± 3.8% and 18.7 ± 4.3% of the total voxels were attributed to cortical and trabecular bone, respectively. For the inferior half, 36.3 ± 6.7% and 24.8 ± 6.4% of the total voxels were attributed to cortical and trabecular bone, respectively. Only the trabecular bone in the superior (*R* ^2^ = 0.546, *p* = 0.0001) and inferior (*R* ^2^ = 0.304, *p* = 0.002) halves showed significant correlations between the number of bone voxels and age (Fig. 3*C*).

### Associations between the amount of bone and age differ between narrow and wide femoral necks

**Fig. 4:**
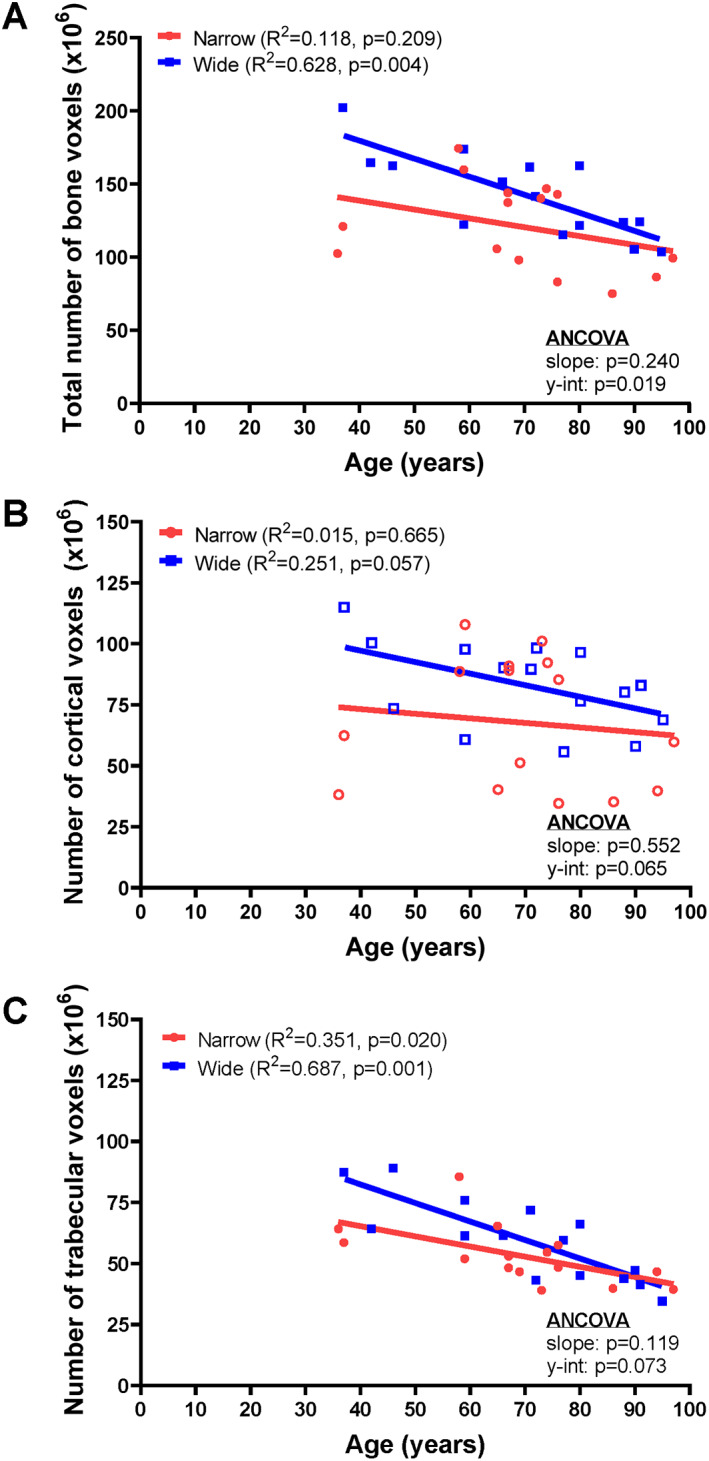
Linear regression analyses show correlations between (*A*) the total amount of bone (number of voxels), (*B*) the amount of cortical bone, and (*C*) the amount of trabecular bone and age for the narrow and wide subgroups. The linear regressions included *n* = 15 for the narrow subgroup and *n* = 15 for the wide subgroup.

Samples were sorted into narrow (*n* = 15) and wide (*n* = 15) subgroups by pseudo‐DXA area. Neither age nor body weight differed between the narrow and wide subgroups (Table 2). Linear regression analyses compared the associations between the number of bone voxels (total, cortical, trabecular) and age for the narrow and wide subgroups (Fig. 4). The wide subgroup showed a significant negative correlation between the total number of bone voxels and age (*R* ^2^ = 0.628, *p* = 0.004), but the narrow subgroup did not (*R* ^2^ = 0.118, *p* = 0.209). The *y*‐intercepts were significantly different (*p* = 0.019, ANCOVA), suggesting the FN ROI was composed of a larger amount of bone tissue for the wide compared with the narrow subgroup at the younger ages of our cohort. The age associations for the cortical (Fig. 4*B*) and trabecular (Fig. 4*C*) tissues were generally consistent with that of the total amount of bone (Fig. 4*A*) with a few exceptions. The wide subgroup showed a significant negative correlation with age for trabecular bone (*p* = 0.001) and borderline significant negative correlation for cortical bone (*p* = 0.057), which explain how the decline in the total amount of bone arose from age‐related declines in both tissue types. In contrast, the narrow subgroup showed a significant negative correlation with age for the trabecular bone only, which may explain why the negative association between total bone voxels and age was not significant. A comparison of the linear regressions between the narrow and wide subgroups showed nonsignificant differences in the *y*‐intercepts; the significance of both the cortical and trabecular regressions were borderline (*p* = 0.065–0.073), suggesting the two tissue types combined accounted for the *y*‐intercept difference in the total amount of bone.

**Table 2:** Comparison of Demographic, Pseudo‐DXA Parameters, and Bone Strength Between Narrow and Wide Subgroups

Parameter	Narrow (*n* = 15^a^)		Wide (*n* = 15)		*t* Test
	Average	Stdev	Average	Stdev	*p* Value
Age (years)	66.93	16.06	71.88	19.02	0.452
Weight (kg)	62.73	23.70	70.68	24.82	0.379
Pseudo‐DXA area (cm^2^)	4.44	0.14	4.82	0.22	**0.0001**
Pseudo‐DXA BMC (10^6^ voxels)	122.65	30.98	139.77	29.70	0.134
Pseudo‐DXA BMD (10^6^ voxels/cm^2^)	27.67	7.11	28.90	5.62	0.601
Fraction of cortical bone	0.539	0.112	0.586	0.068	0.177
Maximum load (N)	2948.2	1134.7	3075.28	897.6	0.739
PYD (mm)	2.52	2.50	3.20	3.69	0.576

*Note*: Data shown as mean ± standard deviation (stdev). Bold values indicate significant differences (*p* < 0.05).

Abbreviations: BMC = bone mineral content; BMD = bone mineral density; PYD = post‐yield deflection.

^a^ The narrow subgroup examined only 14 samples for maximum load and PYD.

### Associations between pseudo‐DXA BMC and the proportion of cortical and trabecular bone differ between narrow and wide femoral necks

**Fig. 5:**
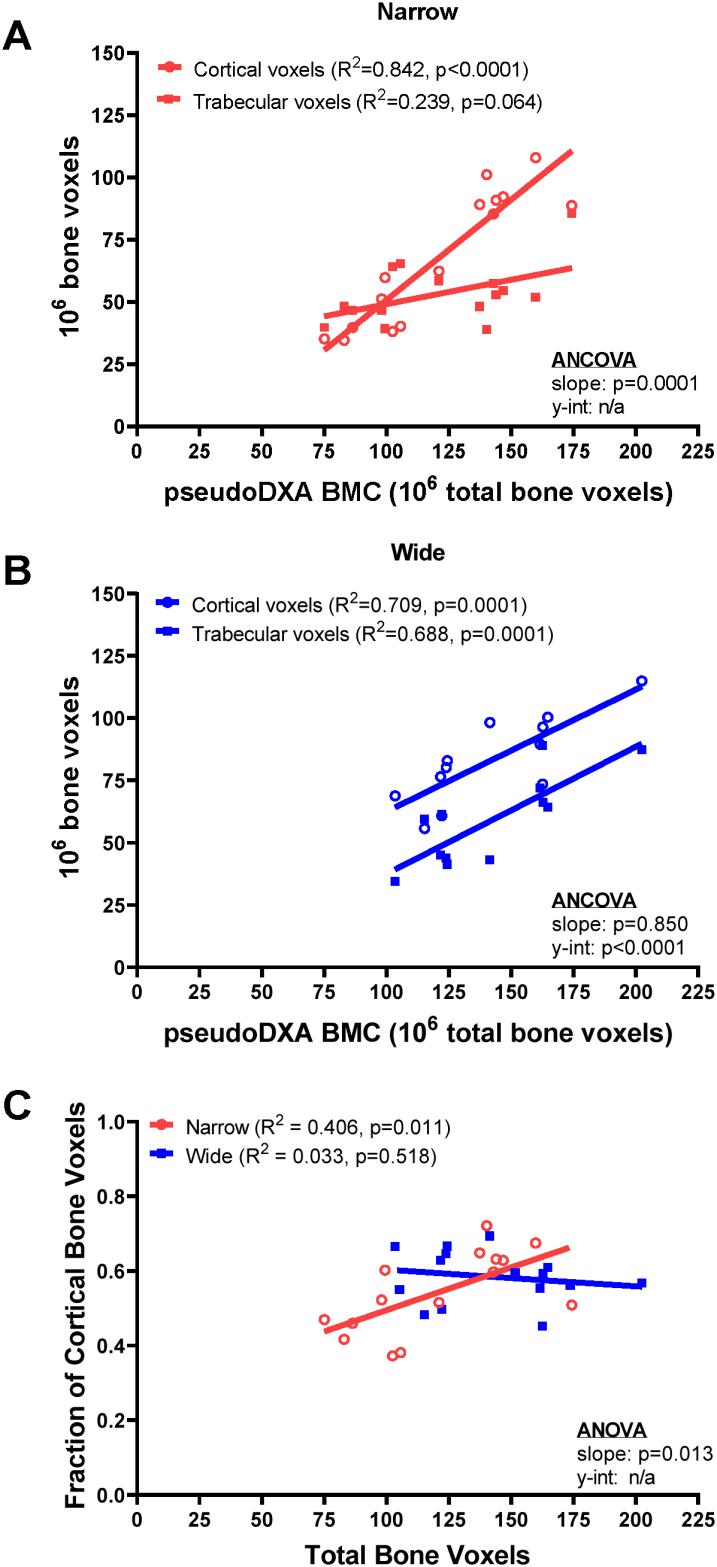
Linear regression between the number of cortical and trabecular bone voxels relative to the total number of voxels shows how variation in bone mineral content (BMC) reflects different proportions of cortical and trabecular bone for the (*A*) narrow and (*B*) wide subgroups. (*C*) The fraction of cortical bone voxels correlated significantly with total number of bone voxels for the narrow but not wide subgroup. The linear regressions included *n* = 15 for the narrow subgroup and *n* = 15 for the wide subgroup.

Pseudo‐DXA BMC (*p* = 0.134) and pseudo‐DXA BMD (*p* = 0.601) did not differ between narrow and wide subgroups (Table 2). Further, the fraction of bone voxels within the FN ROI attributed to cortical bone did not differ between the narrow (0.539 ± 0.112, mean ± SD) and wide (0.587 ± 0.068) subgroups (*p* = 0.177). However, when segmenting total bone voxels into cortical and trabecular voxels (Fig. 5), the narrow subgroup showed significant correlations between pseudo‐DXA BMC and the number of cortical bone voxels (*R* ^2^ = 0.842, *p* < 0.0001) but a weaker association with trabecular bone voxels (*R* ^2^ = 0.239, *p* = 0.064). In contrast, the wide subgroup showed significant associations between pseudo‐DXA BMC and the number of cortical (*R* ^2^ = 0.709, *p* = 0.001) and trabecular (*R* ^2^ = 0.688, *p* = 0.0001) bone voxels. When the number of cortical voxels was expressed as a fraction of total bone voxels, a significant positive correlation was observed between pseudo‐DXA BMC and the fraction of cortical bone voxels for the narrow (*R* ^2^ = 0.406, *p* = 0.011) but not the wide (*R* ^2^ = 0.033, *p* = 0.518) subgroups (ANCOVA, slope *p* = 0.013). The wide subgroup showed a consistent ratio of cortical to trabecular bone (0.59 ± 0.07) for the entire range of pseudo‐DXA BMC values.

### BMD–strength associations depend on external bone size

The fall‐to‐the‐side loading direction resulted in similar distributions of femoral neck and trochanteric fractures for the narrow (3 femoral neck, 11 trochanteric) and wide (4 femoral neck, 11 trochanteric) subgroups. The multivariate linear regression analyses were conducted to identify strength predictors and to determine if the significant predictors differed between narrow and wide subgroups (Table 3). Model 1 predictors included basic information such as age, body weight (BW), and pseudo‐DXA BMD. The adjusted *R* ^2^ was 0.579 when all samples were included in the analysis. When the analyses were conducted by subgroup, adjusted *R* ^2^ values increased to 0.788 for the narrow subgroup but remained relatively modest at 0.491 for the wide subgroup. Replacing pseudo‐DXA BMD with pseudo‐DXA area and pseudo‐DXA BMC (model 2) improved the adjusted *R* ^2^ for both the narrow (adj. *R* ^2^ = 0.892) and wide (adj. *R* ^2^ = 0.537) subgroups. Replacing pseudo‐DXA BMC with the amount of bone in the superior and inferior halves did not appreciably affect the adjusted *R* ^2^ values for either subgroup (model 3). Likewise, breaking the total amount of bone in the superior and inferior halves into cortical and trabecular bone also did not appreciably affect the adjusted *R* ^2^ values for either subgroup (model 4) but did reveal subregions contributing significantly (trabecular bone – inferior half) or borderline significantly (cortical bone – superior and inferior halves) to whole bone strength for the narrow subgroup. Adding PYD, a measure of material brittleness, as a predictor in the regression analyses (model 5) resulted in a large increase in the adjusted *R* ^2^ to 0.706 for the wide subgroup but not the narrow subgroup. The variance inflation factors were generally less than 10 and the models remained significant when individual terms having VIF >10 were removed (data not shown).

**Table 3:** Multivariate Linear Regression Analyses Predicting Whole Bone Strength

Model	All samples (*n* = 29)	Narrow (*n* = 14)	Wide (*n* = 15)
Model 1	*R*_adj_^2^ = 0.579; *p* = 0.001	*R*_adj_^2^ = 0.788; *p* = 0.001	*R*_adj_^2^ = 0.491; *p* = 0.015
Age	−0.215 (0.224)	0.061 (0.726)	−0.508 (0.163)
Log (BW)	−0.305 (0.134)	**−1.159 (0.003)**	−0.022 (0.925)
Pseudo‐DXA BMD	**0.818 (0.003)**	**1.773 (0.000)**	0.311 (0.423)
Model 2	*R*_adj_^2^ = 0.556; *p* = 0.001	*R*_adj_^2^ = 0.892; *p* = 0.001	*R*_adj_^2^ = 0.537; *p* = 0.017
Age	−0.198 (0.289)	0.150 (0.260)	−0.432 (0.227)
Log (BW)	−0.275 (0.193)	**−1.465 (0.000)**	−0.026 (0.906)
Pseudo‐DXA Area	***−0.270 (0.069)***	0.060 (0.552)	−0.385 (0.124)
Pseudo‐DXA BMC	**0.853 (0.005)**	**2.127 (0.000)**	0.473 (0.269)
Model 3	*R*_adj_^2^ = 0.561; *p* = 0.001	*R*_adj_^2^ = 0.923; *p* = 0.001	*R*_adj_^2^ = 0.531; *p* = 0.031
Age	−0.152 (0.422)	0.163 (0.151)	−0.290 (0.453)
Log (BW)	−0.220 (0.306)	**−1.735 (0.001)**	0.011 (0.962)
Pseudo‐DXA area	−0.150 (0.406)	0.020 (0.819)	−0.304 (0.248)
Total voxels superior	**0.707 (0.030)**	**0.663 (0.014)**	0.602 (0.213)
Total voxels inferior	0.137 (0.706)	**1.751 (0.001)**	−0.035 (0.933)
Model 4	*R*_adj_^2^ = 0.536; *p* = 0.001	*R*_adj_^2^ = 0.917; *p* = 0.001	*R*_adj_^2^ = 0.423; *p* = 0.128
Age	−0.247 (0.275)	0.172 (0.242)	−0.016 (0.981)
Log (BW)	−0.208 (0.355)	**−1.589 (0.002)**	0.026 (0.920)
Pseudo‐DXA area	−0.147 (0.430)	−0.066 (0.586)	−0.176 (0.646)
Cortical superior	***0.493 (0.070)***	***0.828 (0.055)***	0.240 (0.481)
Trabecular superior	0.277 (0.307)	0.199 (0.251)	0.788 (0.351)
Cortical inferior	0.125 (0.714)	***1.246 (0.057)***	−0.027 (0.944)
Trabecular inferior	0.006 (0.978)	**0.697 (0.009)**	−0.139 (0.720)
Model 5	*R*_adj_^2^ = 0.628; *p* = 0.001	*R*_adj_^2^ = 0.907; *p* = 0.003	*R*_adj_^2^ = 0.706; *p* = 0.030
Age	−0.237 (0.244)	0.202 (0.235)	−0.332 (0.506)
Log (BW)	−0.062 (0.767)	**−1.510 (0.008)**	0.198 (0.337)
Pseudo‐DXA area	−0.088 (0.602)	−0.131 (0.462)	−0.280 (0.330)
Cortical superior	**0.551 (0.028)**	***0.815 (0.079)***	***0.597 (0.064)***
Trabecular superior	***0.471 (0.074)***	0.224 (0.248)	0.659 (0.288)
Cortical inferior	−0.106 (0.741)	1.151 (0.106)	−0.312 (0.318)
Trabecular inferior	−0.141 (0.506)	**0.686 (0.017)**	−0.338 (0.266)
Sqrt (PYD)	**−0.327 (0.022)**	−0.098 (0.583)	**−0.538 (0.032)**

*Note*: Data shown include the standardized beta coefficients (*p* value). Bold indicates significant relationships, and bold/italics indicates borderline significance.

Abbreviations: BW = body weight; PYD = post‐yield deflection; BMC = bone mineral content; BMD = bone mineral density.

The variables identified as contributing significantly to the prediction of strength differed between the narrow and wide subgroups. Body weight was a consistently strong predictor for the narrow subgroup, as was the number of cortical voxels in the superior and inferior halves and the number of trabecular voxels in the inferior half. For the wide subgroup, significant predictors included the number of cortical voxels in the superior half and PYD.

### Sensitivity analysis

The outcomes of the sensitivity analysis are shown in Supplemental Fig. S1 and Supplemental Table S1. The associations between the number of bone voxels and the number of trabecular voxels and pseudo‐DXA BMC (total number of bone voxels) were consistent when using all data and when excluding the middle 4, 8, and 12 samples (Supplemental Fig. S1). Likewise, the multivariate regression analyses also showed consistent adjusted R‐squared values for the five models, albeit there were changes in the significance of these associations, which was expected as the sample sizes became progressively smaller.

## Discussion

The goals of this study were to understand how variation in bone structure gives rise to variation in BMD and strength and to test whether these associations differ with external bone size. BMD is relied upon clinically to reflect bone structure changes affecting strength and fracture risk.^(^ ^37^, ^38^ ^)^ Although BMD correlates significantly with experimentally determined strength, a critical evaluation of these associations shows a two‐ to threefold variation in strength for a given BMD value.^(^ ^15^, ^31^, ^39^ ^)^ Because half of the individuals who fracture have hip BMD values above the osteoporosis threshold,^(^ ^11^, ^12^, ^13^, ^14^ ^)^ there is a need to better understand how projecting a three‐dimensional structure onto a plane and reducing the bone mineral distribution map within a specified boundary to scalar variables (Fig. 1) affect the association between BMD and strength.^(^ ^37^, ^38^ ^)^ High‐resolution 2D bone mineral distribution maps (pseudo‐DXA images) of the FN ROI were generated from 3D nano‐CT images of unfixed cadaveric proximal femurs to investigate and partition the underlying structure with sufficient resolution to quantify cortical and trabecular architecture. The pseudo‐DXA output parameters correlated significantly with the corresponding parameters measured from a DXA system (Fig. 2). The DXA parameters, the age associations of the pseudo‐DXA parameters, and the BMD–strength association reported herein were consistent with prior studies.^(^ ^30^, ^31^, ^40^ ^)^ Thus, we demonstrated a clinically relevant research tool to investigate the structural determinants of BMD and strength by defining pseudo‐DXA images from 3D nano‐CT volumes of proximal femurs experimentally tested to assess strength.

A major outcome of the current study was finding pseudo‐DXA BMD did not predict strength uniformly across the study cohort. The multivariate regression analysis showed strength was better predicted by dividing the study cohort into subgroups based on external size, allowing for different structure–function associations. Study cohorts are often treated as homogeneous, assuming a single structure–function relationship will identify structural features that predict strength for an entire cohort.^(^ ^41^ ^)^ Many prior studies seeking to identify structural features that differ between subgroups sorted cohorts by fracture status,^(^ ^42^, ^43^, ^44^, ^45^ ^)^ fracture location,^(^ ^46^ ^)^ bone turnover,^(^ ^47^ ^)^ sex,^(^ ^48^, ^49^ ^)^ and race/ethnicity^(^ ^50^ ^)^; in general, these studies assumed a single structure–function relationship existed for each subgroup and that a comparison between subgroups would identify a trait capable of explaining fracture risk for a population. The current study deviated from this approach by testing for more than one structure–function relationship within the cohort. In some prior studies, sorting individuals based on fracture location has successfully identified different structural determinants of fracture risk and bone strength.^(^ ^51^, ^52^, ^53^ ^)^ Our current work builds on these studies by showing different structure–function relationships based on external bone size. Investigating the effects of heterogeneity in relation to bone structure and strength may provide the means for defining multiple biomechanical pathways leading to fracture and moving toward optimal treatment strategies for subgroups based on bone structure.

The significant predictors of strength differed for the narrow and wide subgroups (Table 3). Nearly 80% of the variation in strength of the narrow subgroup was explained by age, body weight, and pseudo‐DXA BMD. Including additional structural or density distribution information in regression models 2 to 5 modestly improved the adjusted R‐squared values, explaining more than 90% of the variation in strength. Thus, simply knowing the amount of bone present was sufficient to predict strength for the narrow subgroup. In contrast, age, body weight, and pseudo‐DXA BMD explained only half the variation in strength for the wide subgroup. Including variables providing greater insight into bone mineral distribution (model 3) or segmentation into cortical and trabecular tissues (model 4) did not improve the adjusted R‐squared values for the wide subgroup. Including PYD, a measure of bone brittleness, in model 5 increased the adjusted *R*‐squared to a value more than 70% for the wide subgroup but did not improve the strength prediction for the narrow subgroup. This outcome suggests material‐level effects may play an important role in the strength of wide femoral necks but not narrow. Thus, unlike the narrow subgroup, measures of the amount of bone may correlate with strength but have limited ability to explain the variation in strength, suggesting proximal femurs in the wide subgroup may undergo a different failure mechanism when overloaded. As such, the wide subgroup appears to depend on structural and material traits differently from the narrow.^(^ ^41^, ^54^ ^)^

DXA parameters have generally been related to bone structure with the intent of understanding the limitations of estimating morphology^(^ ^48^, ^55^, ^56^, ^57^, ^58^, ^59^ ^)^ and strength.^(^ ^19^, ^60^ ^)^ Only a few studies investigated the structural features responsible for the variation in BMD.^(^ ^15^, ^19^, ^20^, ^27^, ^61^ ^)^ Structural variation within the inferior half of the FN was associated with more than 60% of the variation in pseudo‐DXA BMC (Fig. 3), consistent with prior work,^(^ ^61^ ^)^ and most of the BMC variation was attributed to the inferior cortex. This distribution was expected, given the greater thickness of the inferior cortex and the greater density of the compressive trabecular arcade compared with those features within the superior half. The amount of cortical bone comprising the inferior and superior cortices showed nonsignificant (borderline significant for the wide subgroup) associations with age (Fig. 3*C*). Only the amount of trabecular bone showed significant associations with age, whether sorted into superior and inferior halves (Fig. 3*C*) or narrow and wide subgroups (Fig. 4*B, C*). The amount of trabecular bone accounted for less than half of the variation in pseudo‐DXA BMC, which may explain in part why age‐related changes in BMD tend to be relatively small.^(^ ^62^ ^)^

The proportion of cortical to trabecular bone varied within the narrow subgroup (Fig. 5*A*). The ratio of cortical to trabecular bone varies along the length^(^ ^22^, ^27^ ^)^ and around the circumference^(^ ^28^, ^29^ ^)^ of the femoral neck. However, femoral neck morphology has been estimated from hip DXA assuming this ratio is fixed at 0.60.^(^ ^22^, ^23^, ^24^, ^63^ ^)^ This ratio was 0.59 ± 0.07 for the wide subgroup, consistent with these prior studies. However, the narrow subgroup showed a significant association between the cortical/trabecular bone ratio and pseudo‐DXA BMC with individuals having lower pseudo‐DXA BMC showing a lower ratio compared with those at higher pseudo‐DXA BMC. Thus, the proportion of cortical to trabecular bone was not uniform across the study cohort. Assuming DXA images reflect a fixed proportion of cortical and trabecular bone may affect estimates of morphology and strength. In contrast to the wide subgroup, which maintained a consistent proportion of cortical and trabecular bone across pseudo‐DXA BMC values, the narrow subgroup showed a proportionally greater amount of cortical bone at higher pseudo‐DXA BMC values. These associations suggest that narrow and wide femoral necks contribute to strength with different combinations of femoral neck traits.^(^ ^64^ ^)^

This study has several important limitations. The proximal femurs were imaged with DXA using rice to simulate soft tissue, rather than water, because of safety considerations. Although the DXA parameters were not affected by the amount of rice, additional studies are warranted to study the impact of these scanning parameters on DXA BMC and area. The current study focused on the number of bone voxels, deferring the effects of beam scattering and soft tissues, air, and marrow on the pseudo‐DXA image to future studies. Even without consideration of these imaging effects, the association between the number of bone voxels and DXA BMC showed a coefficient of determination of 0.947 (Fig. 2*A*). This strong correlation suggested the variation in DXA BMC primarily reflects the amount of bone present and including more nuanced imaging effects may contribute relatively little to pseudo‐DXA BMC variation. Although pseudo‐DXA area correlated significantly with DXA area, the coefficient of determination (Fig. 2*B*) was lower than those for BMC (Fig. 2*C*) and BMD (Fig. 2*A*). Further, pseudo‐DXA area consistently underestimated the actual DXA area. This discrepancy may be attributed to the fine resolution of the pseudo‐DXA image, which, unlike the actual DXA image, creates well‐defined boundaries between bone and background. Differences in resolution and boundary detection may contribute to the discrepancy between pseudo‐DXA area and DXA area. Anatomical positioning and 3D orientation of the nano‐CT volume were highly repeatable but may contribute additional error. These errors were considered negligible, given the strong correlation between pseudo‐DXA BMD and DXA BMD, and the outcome of the sensitivity analysis, which confirmed subgroup assignment did not meaningfully affect the major outcomes and suggested the overall results of this study are robust. The cadaveric samples were composed of White female donors. Additional studies are needed to test if similar structure–function relationships hold for men and other races/ethnicities. The sample size was sufficient to address the major questions of this study, but additional samples are needed to test how the associations between cortical and trabecular voxels in different regions (superior, inferior) vary with external bone size. Lack of power for these latter analyses may explain the borderline significant differences for the age regressions for cortical (Fig. 4*B*) and trabecular (Fig. 4*C*) bone. There are limitations in the extent to which the age associations shown for cadaveric tests reflect how age‐related changes in bone structure would be reflected in BMD and strength. Examination of longitudinally acquired DXA images are needed to confirm the associations reported herein.

**Fig. 6:**
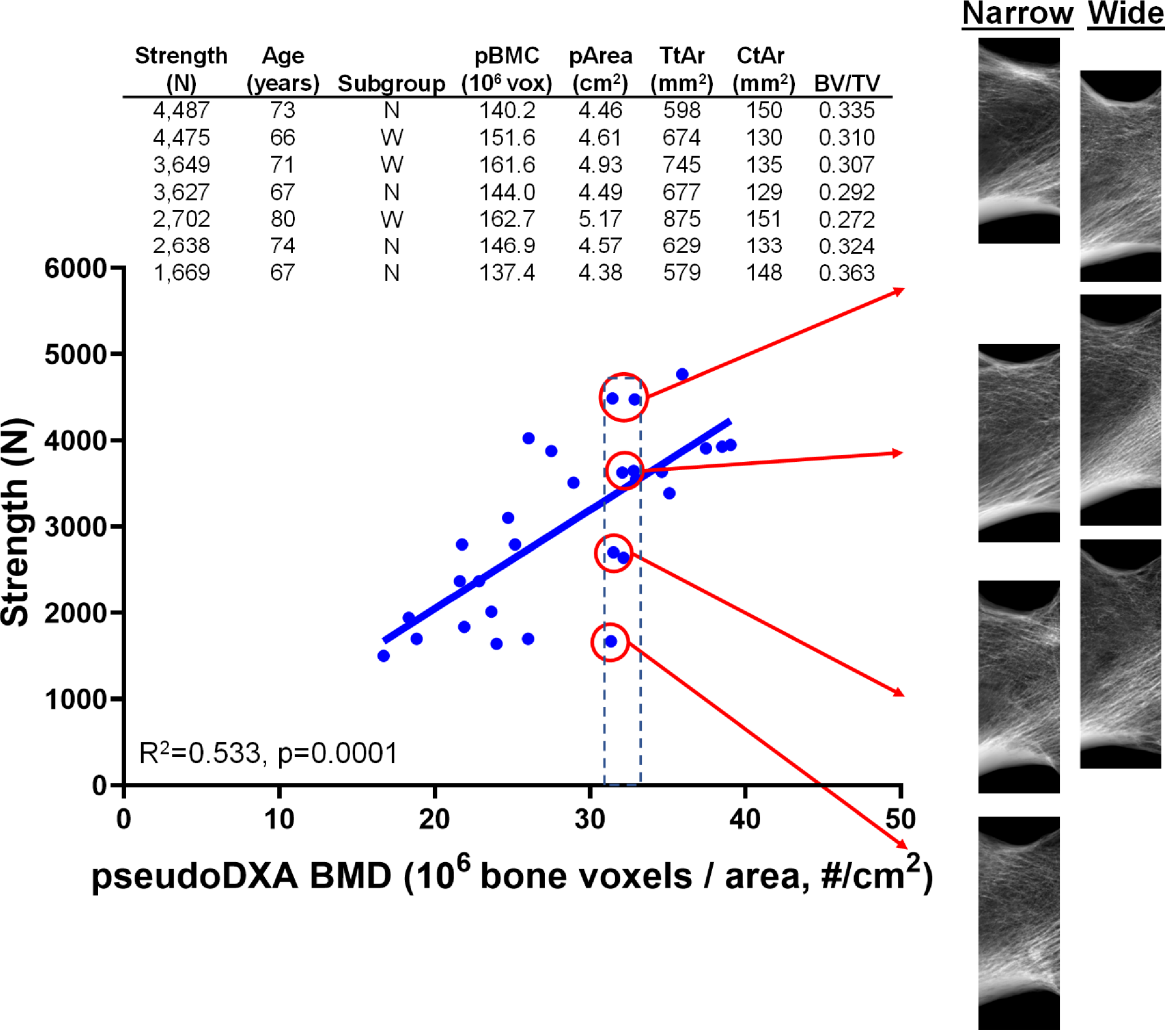
Schematic summarizing how different bone structures give rise to similar bone mineral density (BMD) values, which, in turn, give rise to widely varying strength values. BMC = bone mineral content; Tt.Ar = cross‐sectional total area averaged over region of interest; Ct.Ar = cross‐sectional cortical area averaged over region of interest; BV/TV = bone volume fraction (bone volume divided by total volume contained with the subendosteal boundary).

In conclusion, our data suggested BMD is not uniquely related to the underlying bone structure, and similar BMD values can give rise to substantially different values of bone strength. As illustrated in Fig. 6 and Supplemental Table S2 and Supplemental Fig. S2, varying combinations of cortical and trabecular structures gave rise to relatively similar BMD values, which were, in turn, associated with a twofold variation in strength. This relatively simple consideration of bone heterogeneity demonstrates that associations between bone structure, BMD, and strength vary, leading to significant differences in bone strength in association with bone structure. Basic information available clinically (BMD, BMC, area) appears sufficient to predict strength for the narrow subgroup. However, this limited set of variables did not predict strength accurately for the wide subgroup. Additional studies are needed with larger sample sizes to better understand how variation in structural and material properties leads to variation in the strength of wide proximal femurs.

## Author Contributions

**Karl J Jepsen:** Conceptualization; funding acquisition; investigation; methodology; resources; supervision; validation; writing – original draft; writing – review and editing. **Erin M.R. Bigelow:** Data curation; formal analysis; project administration; supervision; validation; writing – review and editing. **Michael Casden:** Conceptualization; investigation; methodology; validation; writing – review and editing. **Robert W. Goulet:** Data curation; formal analysis; methodology; project administration; software; validation; writing – review and editing. **Kathryn Kennedy:** Data curation; methodology; validation; visualization; writing – review and editing. **Samantha Hertz:** Investigation; methodology; validation; writing – review and editing. **Chandan Kadur:** Investigation; methodology; validation; writing – review and editing. **Bonnie Nolan:** Conceptualization; investigation; methodology; validation; writing – review and editing. **Kerry Richards‐McCullough:** Conceptualization; investigation; methodology; validation; writing – review and editing. **Steffenie Merillat:** Conceptualization; investigation; methodology; validation; writing – review and editing. **Carrie A. Karvonen‐Gutierrez:** Conceptualization; funding acquisition; resources; validation; writing – review and editing. **Gregory Clines:** Conceptualization; investigation; methodology; validation; writing – review and editing. **Todd L Bredbenner:** Conceptualization; formal analysis; methodology; project administration; resources; software; validation; writing – original draft; writing – review and editing.

## Conflicts of Interest

All authors state they have no conflicts of interest.

### Peer Review

The peer review history for this article is available at https://publons.com/publon/10.1002/jbm4.10715.

## Supporting information


**Table S1.** Outcome of sensitivity analysis showing how the adjusted R‐squared values depend on the number of samples removed from the boundary dividing the narrow and wide subgroups when rank ordered by pseudo‐DXA Area. Multivariate regression outcomes are shown for the narrow and wide subgroups when all data was included and when the middle 4 (2 narrow, 2 wide excluded), middle 8 (4 narrow, 4 wide excluded), and middle 12 (6 narrow, 6 wide excluded) samples were removed. Including the outcomes when all data were included is solely for convenience purposes to compare the outcomes of the sensitivity analyses.
**Table S2.** Demographic, biomechanical, and morphological data for the sample cohort ranked by strength. Subgroup (narrow [N], wide [W]) designation is noted.
**Fig. S1.** As part of the sensitivity analysis, the linear regressions shown in Fig. 5 were repeated while excluding the middle 4 (2 narrow, 2 wide), middle 8 (4 narrow, 4 wide) and middle 12 (6 narrow, 6 wide) samples when rank ordered by pseudo‐DXA area. Supplemental Fig. S1A shows the regressions for the narrow subgroup. Supplemental Fig. S1B shows the regressions for the wide subgroup. Supplemental Fig. S1C shows the regressions comparing percent (%) cortical bone voxels as a function of pseudo‐DXA BMC between the narrow and wide subgroups when the middle 2, middle 4, and middle 6 samples of each subgroup were excluded.
**Fig. S2.** Schematic showing the association between strength and pseudo‐DXA BMD and the corresponding pseudo‐DXA images sorted into narrow and wide subgroups. The individual vertical blue dashed boxes highlight samples with roughly similar pseudo‐DXA BMD values but widely varying strength values. The horizontal red dashed box highlights samples with roughly similar strength values but widely varying pseudo‐DXA BMD values. Note the left‐most dashed blue box contains samples only from individuals with narrow femoral necks.

## References

[1] Burge R , Dawson‐Hughes B , Solomon DH , Wong JB , King A , Tosteson A . Incidence and economic burden of osteoporosis‐related fractures in the United States, 2005–2025. J Bone Miner Res. 2007;22(3):465‐475. 17144789 10.1359/jbmr.061113

[2] Brauer CA , Coca‐Perraillon M , Cutler DM , Rosen AB . Incidence and mortality of hip fractures in the United States. JAMA. 2009;302(14):1573‐1579. 19826027 10.1001/jama.2009.1462 PMC4410861

[3] Khosla S , Cauley JA , Compston J , et al. Addressing the crisis in the treatment of osteoporosis: a path forward. J Bone Miner Res. 2016;32(3):424‐430. 28099754 10.1002/jbmr.3074

[4] Vilaca T , Eastell R , Schini M . Osteoporosis in men. Lancet Diabetes Endocrinol. 2022;10(4):273‐283. 35247315 10.1016/S2213-8587(22)00012-2

[5] US Preventive Services Task Force , Curry SJ , Krist AH , et al. Screening for osteoporosis to prevent fractures: US Preventive Services Task Force recommendation statement. JAMA. 2018;319(24):2521‐2531. 29946735 10.1001/jama.2018.7498

[6] Cooper C , Cole ZA , Holroyd CR , et al. Secular trends in the incidence of hip and other osteoporotic fractures. Osteoporos Int. 2011;22(5):1277‐1288. 21461721 10.1007/s00198-011-1601-6 PMC3546313

[7] Cauley JA , Chalhoub D , Kassem AM , Fuleihan GH . Geographic and ethnic disparities in osteoporotic fractures. Nat Rev Endocrinol. 2014;10(6):338‐351. 24751883 10.1038/nrendo.2014.51

[8] Lewiecki EM , Wright NC , Curtis JR , et al. Hip fracture trends in the United States, 2002 to 2015. Osteoporos Int. 2018;29(3):717‐722. 29282482 10.1007/s00198-017-4345-0

[9] Gullberg B , Johnell O , Kanis JA . World‐wide projections for hip fracture. Osteoporos Int. 1997;7(5):407‐413. 9425497 10.1007/pl00004148

[10] Hernlund E , Svedbom A , Ivergard M , et al. Osteoporosis in the European Union: medical management, epidemiology and economic burden. A report prepared in collaboration with the International Osteoporosis Foundation (IOF) and the European Federation of Pharmaceutical Industry Associations (EFPIA). Arch Osteoporos. 2013;8:136. 24113837 10.1007/s11657-013-0136-1 PMC3880487

[11] Siris ES , Chen YT , Abbott TA , et al. Bone mineral density thresholds for pharmacological intervention to prevent fractures. Arch Intern Med. 2004;164(10):1108‐1112. 15159268 10.1001/archinte.164.10.1108

[12] Wainwright SA , Marshall LM , Ensrud KE , et al. Hip fracture in women without osteoporosis. J Clin Endocrinol Metab. 2005;90(5):2787‐2793. 15728213 10.1210/jc.2004-1568

[13] Cranney A , Jamal SA , Tsang JF , Josse RG , Leslie WD . Low bone mineral density and fracture burden in postmenopausal women. CMAJ. 2007;177(6):575‐580. 17846439 10.1503/cmaj.070234 PMC1963365

[14] Tremollieres FA , Pouilles JM , Drewniak N , Laparra J , Ribot CA , Dargent‐Molina P . Fracture risk prediction using BMD and clinical risk factors in early postmenopausal women: sensitivity of the WHO FRAX tool. J Bone Miner Res. 2010;25(5):1002‐1009. 20200927 10.1002/jbmr.12 PMC3112173

[15] Link TM , Vieth V , Langenberg R , et al. Structure analysis of high resolution magnetic resonance imaging of the proximal femur: in vitro correlation with biomechanical strength and BMD. Calcif Tissue Int. 2003;72(2):156‐165. 12370799 10.1007/s00223-001-2132-5

[16] Marshall LM , Lang TF , Lambert LC , et al. Dimensions and volumetric BMD of the proximal femur and their relation to age among older U.S. men. J Bone Miner Res. 2006;21(8):1197‐1206. 16869717 10.1359/jbmr.060506

[17] Luo Y . Bone mineral density averaged over a region of interest on femur is affected by age‐related change of bone geometry. Osteoporos Int. 2018;29(6):1419‐1425. 29508039 10.1007/s00198-018-4461-5

[18] Mittra E , Rubin C , Gruber B , Qin YX . Evaluation of trabecular mechanical and microstructural properties in human calcaneal bone of advanced age using mechanical testing, microCT, and DXA. J Biomech. 2008;41(2):368‐375. 17953972 10.1016/j.jbiomech.2007.09.003

[19] Perilli E , Briggs AM , Kantor S , et al. Failure strength of human vertebrae: prediction using bone mineral density measured by DXA and bone volume by micro‐CT. Bone. 2012;50(6):1416‐1425. 22430313 10.1016/j.bone.2012.03.002

[20] Schmidutz F , Schopf C , Yan SG , Ahrend MD , Ihle C , Sprecher C . Cortical bone thickness of the distal radius predicts the local bone mineral density. Bone Joint Res. 2021;10(12):820‐829. 34927444 10.1302/2046-3758.1012.BJR-2020-0271.R1 PMC8696524

[21] Blake GM , Fogelman I . Technical principles of dual energy X‐ray absorptiometry. Semin Nucl Med. 1997;27(3):210‐228. 9224663 10.1016/s0001-2998(97)80025-6

[22] Kuiper JW , Van Kuijk C , Grashuis JL . Distribution of trabecular and cortical bone related to geometry. A quantitative computed tomography study of the femoral neck. Invest Radiol. 1997;32(2):83‐89. 9039579 10.1097/00004424-199702000-00002

[23] Beck TJ . Extending DXA beyond bone mineral density: understanding hip structure analysis. Curr Osteoporos Rep. 2007;5(2):49‐55. 17521505 10.1007/s11914-007-0002-4

[24] Danielson ME , Beck TJ , Karlamangla AS , et al. A comparison of DXA and CT based methods for estimating the strength of the femoral neck in post‐menopausal women. Osteoporos Int. 2013;24(4):1379‐1388. 22810918 10.1007/s00198-012-2066-y PMC3606278

[25] Wolff J . The law of bone remodelling. Berlin: Springer; 1986 p 1892.

[26] Nazarian A , Muller J , Zurakowski D , Muller R , Snyder BD . Densitometric, morphometric and mechanical distributions in the human proximal femur. J Biomech. 2007;40(11):2573‐2579. 17258221 10.1016/j.jbiomech.2006.11.022

[27] Zebaze RM , Jones A , Welsh F , Knackstedt M , Seeman E . Femoral neck shape and the spatial distribution of its mineral mass varies with its size: clinical and biomechanical implications. Bone. 2005;37(2):243‐252. 15939679 10.1016/j.bone.2005.03.019

[28] Bell KL , Garrahan N , Kneissel M , et al. Cortical and cancellous bone in the human femoral neck: evaluation of an interactive image analysis system. Bone. 1996;19(5):541‐548. 8922655 10.1016/s8756-3282(96)00245-1

[29] Bell KL , Loveridge N , Power J , et al. Structure of the femoral neck in hip fracture: cortical bone loss in the inferoanterior to superoposterior axis. J Bone Miner Res. 1999;14(1):111‐119. 9893072 10.1359/jbmr.1999.14.1.111

[30] Jepsen KJ , Kozminski A , Bigelow EM , et al. Femoral neck external size but not aBMD predicts structural and mass changes for women transitioning through menopause. J Bone Miner Res. 2017;32(6):1218‐1228. 28084657 10.1002/jbmr.3082 PMC5466474

[31] Rezaei A , Dragomir‐Daescu D . Femoral strength changes faster with age than BMD in both women and men: a biomechanical study. J Bone Miner Res. 2015;30(12):2200‐2206. 26096829 10.1002/jbmr.2572

[32] Bouxsein ML , Boyd SK , Christiansen BA , Guldberg RE , Jepsen KJ , Muller R . Guidelines for assessment of bone microstructure in rodents using micro‐computed tomography. J Bone Miner Res. 2010;25(7):1468‐1486. 20533309 10.1002/jbmr.141

[33] Luo Y . Image‐based multilevel biomechanical modeling for fall‐induced hip fracture. New York, NY: Springer Science + Business Media; 2016.

[34] Patton DM , Bigelow EMR , Schlecht SH , Kohn DH , Bredbenner TL , Jepsen KJ . The relationship between whole bone stiffness and strength is age and sex dependent. J Biomech. 2019;83:125‐133. 30527634 10.1016/j.jbiomech.2018.11.030 PMC6338331

[35] Cody DD , Gross GJ , Hou FJ , Spencer HJ , Goldstein SA , Fyhrie DP . Femoral strength is better predicted by finite element models than QCT and DXA. J Biomech. 1999;32(10):1013‐1020. 10476839 10.1016/s0021-9290(99)00099-8

[36] Nawathe S , Yang H , Fields AJ , Bouxsein ML , Keaveny TM . Theoretical effects of fully ductile versus fully brittle behaviors of bone tissue on the strength of the human proximal femur and vertebral body. J Biomech. 2015;48(7):1264‐1269. 25828400 10.1016/j.jbiomech.2015.02.066

[37] Bouxsein ML , Karasik D . Bone geometry and skeletal fragility. Curr Osteoporos Rep. 2006;4(2):49‐56. 16822403 10.1007/s11914-006-0002-9

[38] Bredbenner TL , Mason RL , Havill LM , Orwoll ES , Nicolella DP , Osteoporotic Fractures in Men Study . Fracture risk predictions based on statistical shape and density modeling of the proximal femur. J Bone Miner Res. 2014;29(9):2090‐2100. 24692132 10.1002/jbmr.2241 PMC4357175

[39] Nawathe S , Akhlaghpour H , Bouxsein ML , Keaveny TM . Microstructural failure mechanisms in the human proximal femur for sideways fall loading. J Bone Miner Res. 2014;29(2):507‐515. 23832419 10.1002/jbmr.2033

[40] Huber MB , Carballido‐Gamio J , Bauer JS , et al. Proximal femur specimens: automated 3D trabecular bone mineral density analysis at multidetector CT‐correlation with biomechanical strength measurement. Radiology. 2008;247(2):472‐481. 18430879 10.1148/radiol.2472070982

[41] Loundagain LL , Bredbenner TL , Jepsen KJ , Edwards WB . Bringing mechanical context to image‐based measurements of bone integrity. Curr Osteoporos Rep. 2021;19(5):542‐552. 34269975 10.1007/s11914-021-00700-z

[42] Faulkner KG , Cummings SR , Black D , Palermo L , Gluer CC , Genant HK . Simple measurement of femoral geometry predicts hip fracture: the study of osteoporotic fractures. J Bone Miner Res. 1993;8(10):1211‐1217. 8256658 10.1002/jbmr.5650081008

[43] Gluer CC , Cummings SR , Pressman A , et al. Prediction of hip fractures from pelvic radiographs: the Study of Osteoporotic Fractures. The Study of Osteoporotic Fractures Research Group. J Bone Miner Res. 1994;9(5):671‐677. 8053396 10.1002/jbmr.5650090512

[44] Karlsson KM , Sernbo I , Obrant KJ , Redlund‐Johnell I , Johnell O . Femoral neck geometry and radiographic signs of osteoporosis as predictors of hip fracture. Bone. 1996;18(4):327‐330. 8726389 10.1016/8756-3282(96)00004-x

[45] Villamor E , Monserrat C , Del Rio L , Romero‐Martin JA , Ruperez MJ . Prediction of osteoporotic hip fracture in postmenopausal women through patient‐specific FE analyses and machine learning. Comput Methods Programs Biomed. 2020;193:105484. 32278980 10.1016/j.cmpb.2020.105484

[46] Duboeuf F , Hans D , Schott AM , et al. Different morphometric and densitometric parameters predict cervical and trochanteric hip fracture: the EPIDOS study. J Bone Miner Res. 1997;12(11):1895‐1902. 9383694 10.1359/jbmr.1997.12.11.1895

[47] Shieh A , Greendale GA , Cauley JA , Karlamangla AS . The association between fast increase in bone turnover during the menopause transition and subsequent fracture. J Clin Endocrinol Metab. 2020;105(4):e1440‐e1448. 31840764 10.1210/clinem/dgz281 PMC7067542

[48] Beck TJ , Ruff CB , Scott WW , Plato CC , Tobin JD , Quan CA . Sex differences in geometry of the femoral neck with aging: a structural analysis of bone mineral data. Calcif Tissue Int. 1992;50(1):24‐29. 1739866 10.1007/BF00297293

[49] Duan Y , Beck TJ , Wang XF , Seeman E . Structural and biomechanical basis of sexual dimorphism in femoral neck fragility has its origins in growth and aging. J Bone Miner Res. 2003;18(10):1766‐1774. 14584886 10.1359/jbmr.2003.18.10.1766

[50] Zhang F , Tan LJ , Lei SF , Deng HW . The differences of femoral neck geometric parameters: effects of age, gender and race. Osteoporos Int. 2010;21(7):1205‐1214. 19802512 10.1007/s00198-009-1057-0 PMC2921984

[51] Szulc P , Duboeuf F , Schott AM , Dargent‐Molina P , Meunier PJ , Delmas PD . Structural determinants of hip fracture in elderly women: re‐analysis of the data from the EPIDOS study. Osteoporos Int. 2006;17(2):231‐236. 15983728 10.1007/s00198-005-1980-7

[52] Pulkkinen P , Eckstein F , Lochmuller EM , Kuhn V , Jamsa T . Association of geometric factors and failure load level with the distribution of cervical vs. trochanteric hip fractures. J Bone Miner Res. 2006;21(6):895‐901. 16753020 10.1359/jbmr.060305

[53] Pulkkinen P , Gluer CC , Jamsa T . Investigation of differences between hip fracture types: a worthy strategy for improved risk assessment and fracture prevention. Bone. 2011;49(4):600‐604. 21807130 10.1016/j.bone.2011.07.022

[54] Bigelow EM , Patton DM , Ward FS , et al. External bone size is a key determinant of strength‐decline trajectories of aging male radii. J Bone Miner Res. 2019;34(5):825‐837. 30715752 10.1002/jbmr.3661 PMC6536328

[55] Ruff CB , Hayes WC . Bone‐mineral content in the lower limb. Relationship to cross‐sectional geometry. J Bone Joint Surg Am. 1984;66(7):1024‐1031. 6480631

[56] Martin RB , Burr DB . Non‐invasive measurement of long bone cross‐sectional moment of inertia by photon absorptiometry. J Biomech. 1984;17(3):195‐201. 6736056 10.1016/0021-9290(84)90010-1

[57] Beck TJ , Ruff CB , Warden KE , Scott WW Jr , Rao GU . Predicting femoral neck strength from bone mineral data. A structural approach. Invest Radiol. 1990;25(1):6‐18. 2298552 10.1097/00004424-199001000-00004

[58] Beck TJ , Ruff CB , Mourtada FA , et al. Dual‐energy X‐ray absorptiometry derived structural geometry for stress fracture prediction in male U.S. Marine Corps recruits. J Bone Miner Res. 1996;11(5):645‐653. 9157779 10.1002/jbmr.5650110512

[59] Augat P , Reeb H , Claes LE . Prediction of fracture load at different skeletal sites by geometric properties of the cortical shell. J Bone Miner Res. 1996;11(9):1356‐1363. 8864911 10.1002/jbmr.5650110921

[60] Rivadeneira F , Zillikens MC , De Laet CE , et al. Femoral neck BMD is a strong predictor of hip fracture susceptibility in elderly men and women because it detects cortical bone instability: the Rotterdam study. J Bone Miner Res. 2007;22(11):1781‐1790. 17638578 10.1359/jbmr.070712

[61] Yoshikawa T , Turner CH , Peacock M , et al. Geometric structure of the femoral neck measured using dual‐energy x‐ray absorptiometry. J Bone Miner Res. 1994;9(7):1053‐1064. 7942152 10.1002/jbmr.5650090713

[62] Hui SL , Zhou L , Evans R , et al. Rates of growth and loss of bone mineral in the spine and femoral neck in white females. Osteoporos Int. 1999;9(3):200‐205. 10450407 10.1007/s001980050137

[63] Danielson ME , Beck TJ , Lian Y , et al. Ethnic variability in bone geometry as assessed by hip structure analysis: findings from the hip strength across the menopausal transition study. J Bone Miner Res. 2013;28(4):771‐779. 23044816 10.1002/jbmr.1781 PMC3586935

[64] Epelboym Y , Gendron RN , Mayer J , et al. The inter‐individual variation in femoral neck width is associated with the acquisition of predictable sets of morphological and tissue‐quality traits and differential bone loss patterns. J Bone Miner Res. 2012;27(7):1501‐1510. 22461103 10.1002/jbmr.1614

