# Supplementary material for: Associations Among Hip Structure, Bone Mineral Density, and Strength Vary With External Bone Size in White Women
Source: JBMR Plus. 2023 Jan 16;7(3):e10715. doi: 10.1002/jbm4.10715 (PMC10020918; doi:10.1002/jbm4.10715)
Supplement: Supplementary file 1 — Table S1. Outcome of sensitivity analysis showing how the adjusted R‐squared values depend on the number of samples removed from the boundary dividing the narrow and wide subgroups when rank ordered by pseudo‐DXA Area. Multivariate regression outcomes are shown for the narrow and wide subgroups when all data was included and when the middle 4 (2 narrow, 2 wide excluded), middle 8 (4 narrow, 4 wide excluded), and middle 12 (6 narrow, 6 wide excluded) samples were removed. Including the outcomes when all data were included is solely for convenience purposes to compare the outcomes of the sensitivity analyses. Table S2. Demographic, biomechanical, and morphological data for the sample cohort ranked by strength. Subgroup (narrow [N], wide [W]) designation is noted. Fig. S1. As part of the sensitivity analysis, the linear regressions shown in Fig. 5 were repeated while excluding the middle 4 (2 narrow, 2 wide), middle 8 (4 narrow, 4 wide) and middle 12 (6 narrow, 6 wide) samples when rank ordered by pseudo‐DXA area. Supplemental Fig. S1A shows the regressions for the narrow subgroup. Supplemental Fig. S1B shows the regressions for the wide subgroup. Supplemental Fig. S1C shows the regressions comparing percent (%) cortical bone voxels as a function of pseudo‐DXA BMC between the narrow and wide subgroups when the middle 2, middle 4, and middle 6 samples of each subgroup were excluded. Fig. S2. Schematic showing the association between strength and pseudo‐DXA BMD and the corresponding pseudo‐DXA images sorted into narrow and wide subgroups. The individual vertical blue dashed boxes highlight samples with roughly similar pseudo‐DXA BMD values but widely varying strength values. The horizontal red dashed box highlights samples with roughly similar strength values but widely varying pseudo‐DXA BMD values. Note the left‐most dashed blue box contains samples only from individuals with narrow femoral necks. [file JBM4-7-e10715-s001.docx]

**Supplemental Tables and Figures**

**Table 1.** Outcome of sensitivity analysis showing how the adjusted R-squared values depend on the number of samples removed from the boundary dividing the narrow and wide subgroups when rank-ordered by pseudoDXA Area. Multivariate regression outcomes are shown for the narrow and wide subgroups when all data was included and when the middle 4 (2 narrow, 2 wide excluded), middle 8 (4 narrow, 4 wide excluded), and middle 12 (6 narrow, 6 wide excluded) samples were removed. Including the outcomes when all data were included is solely for convenience purposes to compare the outcomes of the sensitivity analyses.

Bold parameters indicate significant predictors of strength (p<0.05). Bold italic font indicate borderline significant predictors of strength (0.05<p<0.10). Note that models 4 and 5 could not be performed because the number of parameters was too large for the sample size.

BW=body weight, BMD=bone mineral density, BMC=bone mineral content, PYD=post-yield deflection,

FN=femoral neck, Ct-sup=number of cortical voxels in the superior half, Ct-inf=number of cortical voxels in the inferior half, Tb-sup=number of trabecular voxels in the superior half, Tb-inf=number of trabecular voxels in the inferior half, Ttl-sup=total number of bone voxels (cortical + trabecular) in the superior half, Ttl-inf=total number of bone voxels in the inferior half

| **Narrow Subgroup** | | | |
| --- | --- | --- | --- |
| **All data included (n=14)** | **Middle 2 excluded (n=12)** | **Middle 4 excluded (n=10)** | **Middle 6 excluded (n=8)** |
| **Model 1:** adjR^2^= 0.788, p=0.001  Age, **logBW, BMD** | **Model 1:** adjR^2^= 0.763, p=0.002  Age, **logBW, BMD** | **Model 1:** adjR^2^= 0.719, p=0.0.013  Age, **logBW, BMD** | **Model 1:** adjR^2^= 0.700, p=0.052  Age, ***logBW,* BMD** |
| **Model 2:** adjR^2^= 0.892, p=0.001  Age, **logBW,** FN Area, **FN BMC** | **Model 2:** adjR^2^= 0.874, p=0.001  Age, **logBW,** FN Area, **FN BMC** | **Model 2:** adjR^2^= 0.940, p=0.001  ***Age,*** **logBW**, FN Area, **FN BMC** | **Model 2:** adjR^2^= 0.950, p=0.008  Age, **logBW**, FN Area, **FN BMC** |
| **Model 3:** adjR^2^= 0.923, p=0.001  Age, **logBW,** FN Area,  **Ttl Sup,** **TTL inf** | **Model 3:** adjR^2^= 0.905, p=0.001  Age, **logBW,** FN Area,  ***Ttl Sup,*** **TTL inf** | **Model 3:** adjR^2^= 0.950, p=0.002  Age, **logBW,** FN Area,  **Ttl Sup, TTL inf** | **Model 3:** adjR^2^= 0.934, p=0.047  Age, **logBW,** FN Area,  Ttl Sup**, *TTL inf*** |
| **Model 4:** adjR^2^= 0.917, p=0.001  Age, **logBW,** FN Area,  ***Ct-sup,*** Tb-sup, ***Ct-inf,*** **Tb-inf** | **Model 4:** adjR^2^= 0.893, p=0.011  Age, **logBW,** FN Area,  Ct-sup, Tb-sup, Ct-inf, **Tb-inf** | **Model 4:** adjR^2^= 0.922, p=0.059  Age, **logBW,** FN Area,  Ct-sup, Tb-sup, Ct-inf, Tb-inf | **Model 4: n/a** |
| **Model 5:** adjR^2^= 0.907 p=0.003  Age, **logBW,** FN Area,  ***Ct-sup,*** Tb-sup, Ct-inf, **Tb-inf,** sqrt(PYD) | **Model 5:** adjR^2^= 0.869, p=0.042  Age, **logBW,** FN Area,  Ct-sup, Tb-sup, Ct-inf, ***Tb-inf,*** sqrt(PYD) | **Model 5:** adjR^2^= 0.912, p=0.214  Age, logBW, FN Area,  Ct-sup, Tb-sup, Ct-inf, Tb-inf, sqrt(PYD) | **Model 5: n/a** |

| **Wide Subgroup** | | | |
| --- | --- | --- | --- |
| **All data included (n=15)** | **Middle 2 excluded (n=13)** | **Middle 4 excluded (n=11)** | **Middle 6 excluded (n=9)** |
| **Model 1**: adjR^2^= 0.491, p=0.015  **Age**, logBW, BM | **Model 1:** adjR^2^= 0.593, p=0.011  **Age**, logBW, BMD | **Model 1:** adjR^2^= 0.560, p=0.033  Age, logBW, BMD | **Model 1:** adjR^2^= 0.719, p=0.025  Age, logBW, BMD |
| **Model 2:** adjR^2^= 0.537, p=0.017  Age, logBW, FN Area, FN BMC | **Model 2:** adjR^2^= 0.553, p=0.030  ***Age,*** logBW, FN Area, FN BMC | **Model 2:** adjR^2^= 0.498, p=0.085  Age, logBW, FN Area, FN BMC | **Model 2:** adjR^2^= 0.795, p=0.029  Age, logBW, FN Area, FN BMC |
| **Model 3:** adjR^2^= 0.531, p=0.031  Age, logBW, FN Area,  Ttl Sup, TTL inf | **Model 3:** adjR^2^= 0.491, p=0.075  Age, logBW, FN Area,  Ttl Sup, TTL inf | **Model 3:** adjR^2^= 0.406, p=0.183  Age, logBW, FN Area,  Ttl Sup, TTL inf | **Model 3:** adjR^2^= 0.738, p=0.096  Age, logBW, FN Area,  Ttl Sup, TTL inf |
| **Model 4:** adjR^2^= 0.423, p=0.128  Age, logBW, FN Area,  Ct-sup, Tb-sup, Ct-inf, Tb-inf | **Model 4:** adjR^2^= 0.356, p=0.240  Age, logBW, FN Area,  Ct-sup, Tb-sup, Ct-inf, Tb-inf | **Model 4:** adjR^2^= 0.088, p=0.503  Age, logBW, FN Area,  Ct-sup, Tb-sup, Ct-inf, Tb-inf | **Model 4: n/a** |
| **Model 5:** adjR^2^= 0.706, p=0.030  **Age, logBW,** FN Area,  ***Ct-sup***, Tb-sup, Ct-inf, Tb-inf, **sqrt(PYD)** | **Model 5:** adjR^2^= 0.896, p=0.011  **Age, logBW,** FN Area,  Ct-sup, Tb-sup, ***Ct-inf,*** Tb-inf, **sqrt(PYD)** | **Model 5:** adjR^2^= 0.880, p=0.093  Age, logBW, FN Area,  Ct-sup, Tb-sup, Ct-inf, Tb-inf, **sqrt(PYD)** | **Model 5: n/a** |

**Figure S1.** As part of the sensitivity analysis, the linear regressions shown in Figure 5 were repeated while excluding the middle 4 (2 narrow, 2 wide), middle 8 (4 narrow, 4 wide) and middle 12 (6 narrow, 6 wide) samples when rank ordered by pseudoDXA area. Figure S1a shows the regressions for the narrow subgroup. Figure S1b shows the regressions for the wide subgroup. Figure S1c shows the regressions comparing percent (%) cortical bone voxels as a function of pseudoDXA BMC between the narrow and wide subgroups when the middle 2, middle 4, and middle 6 samples of each subgroup were excluded.

**S1a.**

**S1b.**

**S1c.**

**Table 2.** Demographic, biomechanical, and morphological data for the sample cohort ranked by strength. Subgroup (narrow (N), wide (W)) designation is noted.

Abbreviations:

BMC = bone mineral content

BMD = bone mineral density

Ct.Ar = cortical area (average across all cross-sections)

pDXA = pseudoDXA

Strength = maximum load

Tb.BV/TV = trabecular bone volume fraction

Tt.Ar = total area (average across all cross-sections)

vox = bone voxels

**Figure S2.** Schematic showing the association between strength and pseudoDXA BMD and the corresponding pseudoDXA images sorted into narrow and wide subgroups. The individual vertical blue dashed boxes highlight samples with roughly similar pseudoDXA BMD values but widely varying strength values. The horizontal red dashed box highlights samples with roughly similar strength values but widely varying pseudoDXA BMD values. Note the left-most dashed blue box contains samples only from individuals with narrow femoral necks.
